# Hemerythrin E3 Ubiquitin Ligases as Negative Regulators of Iron Homeostasis in Plants

**DOI:** 10.3389/fpls.2019.00098

**Published:** 2019-02-13

**Authors:** Jorge Rodríguez-Celma, Hsuan Chou, Takanori Kobayashi, Terri A. Long, Janneke Balk

**Affiliations:** ^1^Department of Biological Chemistry, John Innes Centre, Norwich, United Kingdom; ^2^School of Biological Sciences, University of East Anglia, Norwich, United Kingdom; ^3^Department of Plant and Microbial Biology, North Carolina State University, Raleigh, NC, United States; ^4^Research Institute for Bioresources and Biotechnology, Ishikawa Prefectural University, Nonoichi, Japan

**Keywords:** micronutrient, iron deficiency, bHLH, FBXL5, zinc finger, sensor

## Abstract

Iron (Fe) is an essential nutrient for plants, but at the same time its redox properties can make it a dangerous toxin inside living cells. Homeostasis between uptake, use and storage of Fe must be maintained at all times. A small family of unique hemerythrin E3 ubiquitin ligases found in green algae and plants play an important role in avoiding toxic Fe overload, acting as negative regulators of Fe homeostasis. Protein interaction data showed that they target specific transcription factors for degradation by the 26S proteasome. It is thought that the activity of the E3 ubiquitin ligases is controlled by Fe binding to the N-terminal hemerythrin motifs. Here, we discuss what we have learned so far from studies on the HRZ (Hemerythrin RING Zinc finger) proteins in rice, the homologous BTS (BRUTUS) and root-specific BTSL (BRUTUS-LIKE) in Arabidopsis. A mechanistic model is proposed to help focus future research questions towards a full understanding of the regulatory role of these proteins in Fe homeostasis in plants.

## Introduction

Plants are stationary, therefore the ability to detect environmental stimuli, interpret them and activate proper physiological responses is crucial for survival, growth, and development. Plants rely on different sensors such as cell receptors to detect and respond to light, hormones, abiotic/biotic stimuli and nutrients. Many of the molecular mechanisms and pathways involved in sensing and responding to these environmental cues have been well studied (Galvão and Fankhauser, 2015; Larrieu and Vernoux, 2015; Ranty et al., 2016). In contrast, very little is known about how plants sense micronutrients such as iron (Fe), zinc (Zn), manganese, copper, and boron. In this review, we focus on a small family of proteins that have been proposed to function as the elusive Fe sensors in plants.

Plants are very efficient in mining the soil for Fe, even though Fe is mostly insoluble in its oxidised form. The uptake of Fe, and the expression of many genes involved in this process, is tightly regulated in line with the requirement of Fe for new growth and photosynthesis (Kobayashi and Nishizawa, 2012; Connorton et al., 2017). It is therefore important to constantly monitor the Fe status, linked to signalling pathways to balance supply and demand.

The molecular mechanisms for Fe sensing and signalling are very diverse in bacteria, fungi and animals (Kobayashi and Nishizawa, 2014; Outten, 2017). Regardless of the differences, Fe sensor systems all require direct binding of Fe or Fe-containing prosthetic groups (Fe-S clusters and heme) which then bring about changes in transcription, translation or protein turnover.

**FIGURE 1:**
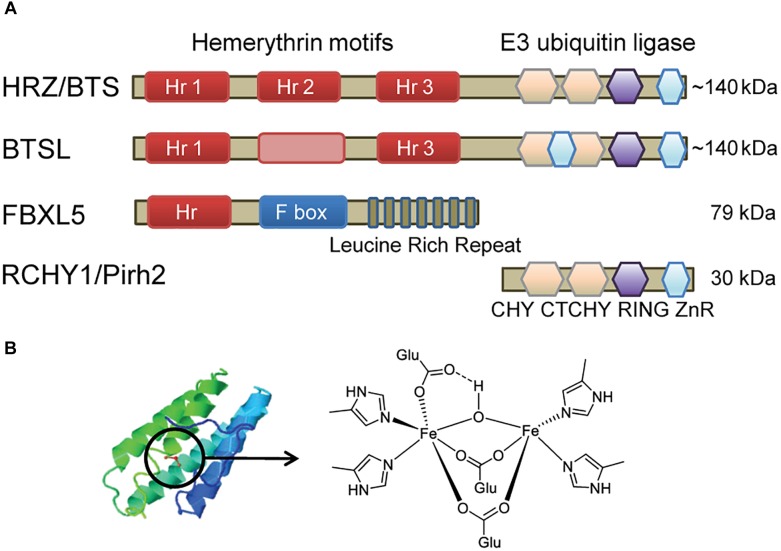
Diagram depicting the main features of hemerythrin E3-ligases. **(A)** Domain organisation of HRZ/BTS/BTSL proteins in plants and algae, compared to homologous proteins in animals. The pink box in BTSL represents a degenerate hemerythrin (Hr) motif with a predicted alpha-helical bundle but lacking the Fe-binding histidine and glutamate residues. The E3 ubiquitin ligase domain has a sequence of different zinc fingers, including CHY-type, CTCHY-type, RING-type and a zinc ribbon (ZnR). **(B)** Typical alpha-helical structure of Hr proteins and the structure of the di-iron centre (Fe-O-Fe) in FBXL5 (Ruiz and Bruick, 2014).

The mammalian protein FBXL5 (F-BoX and Leucine-rich repeat 5) regulates Fe homeostasis by targeting IRP2 (Iron Regulated Protein 2) for degradation (Salahudeen et al., 2009; Vashisht et al., 2009; Thompson et al., 2012). FBXL5 consists of an N-terminal hemerythrin (Hr) motif that binds a redox-active di-iron centre, an F-box domain that forms part of a multi-subunit E3 ubiquitin ligase, and a leucine-rich repeat for protein interactions (Figure 1A). When Fe is bound, the Hr domain is folded and the FBXL5-E3 ligase complex interacts with IRP2, promoting its degradation. When Fe is low, the Hr domain unfolds and FBXL5 is degraded, thus leaving IRP2 intact to stabilise transcripts encoding Fe uptake proteins. At the same time, IRP2 binding blocks the translation of the Fe storage protein ferritin. FBXL5 is also stabilised by oxygen, but the mechanism is not fully understood (Ruiz and Bruick, 2014). Recently, proteins with distant homology to FBXL5 have been identified in plants (Kobayashi et al., 2013). Here, we present relevant data about this novel family of putative Fe sensors, including the HRZ, BTS, and BTSL proteins, and their involvement in controlling Fe accumulation in plants.

## Phylogeny and Protein Motifs

The HRZ/BTS/BTSL proteins are found throughout the green lineage. They are characterised by 2 – 3 Hr motifs at the N-terminus and a RING-type E3 ubiquitin ligase at the C-terminal end (Figure 1A). This unique combination of protein domains is not found in other kingdoms of life (Matthiadis and Long, 2016). The small gene family is represented by one copy in the green alga *Chlamydomonas reinhardtii* (Urzica et al., 2012), two *HRZ* genes in rice, and three genes – *BTS*, *BTSL1*, and *BTSL2* – in Arabidopsis (Kobayashi et al., 2013). Phylogenetic analysis shows that the genes can be divided in two clades; *HRZ*/*BTS* “sensu stricto” are found in all green organisms, whereas the *BTSL* genes are only present in dicotyledon species (bioRxiv: Rodríguez-Celma et al., 2017).

The N-terminus with Hr motifs makes up two-thirds of the ∼140 kDa protein. Hr motifs typically form a bundle of four α-helices, with the di-iron cofactor in the middle bound by histidines and acidic residues. The two Fe ions are bridged by an oxygen or hydroxyl group (Figure 1B; Ruiz and Bruick, 2014). HRZ/BTS proteins have 3 Hr motifs, but BTSL proteins only retain the 1st and 3rd motif. Instead of the 2nd Hr motif, a predicted α-helical bundle is found but not the residues for Fe binding. HRZ2 in rice was initially annotated with one Hr motif corresponding to the 3rd motif in HRZ1 (Kobayashi et al., 2013), however, the open reading frame extends 5′ to reveal another 2 Hr motifs. Thus, 2 or 3 Fe-binding Hr motifs are found in all HRZ/BTS/BTSL proteins and are likely to be critical for their function in plants, in contrast to a single N-terminal Hr motif in the mammalian FBXL5 protein.

E3 ubiquitin ligases form a large family of proteins, with more than 1400 family members in Arabidopsis (Lee and Kim, 2011). By forming specific protein interactions, E3 ligases ubiquitinate target proteins, followed, in most cases, by proteasomal degradation. The E3 ligase domain of HRZ/BTS/BTSL has striking amino acid similarity (∼57%) with human/mouse RCHY1 (also called Pirh2), which belongs to a small subfamily of RING-type E3 ubiquitin ligases (Figure 1A). NMR structures of the three subdomains of RCHY1 revealed 9 Zn-binding sites in different types of Zn fingers: the CHY-type, the CTCHY-type and the RING-type that interacts with the E2 ubiquitin-conjugating enzyme (Sheng et al., 2008). Protein motif searches identified a rubredoxin motif at the very end of the protein, but the NMR structure showed Zn binding rather than Fe. The Zn-binding cysteine and histidine residues are fully conserved in plant HRZ/BTS proteins (Matthiadis and Long, 2016), but an additional Zn-finger motif is present in BTSL proteins (Figure 1A). Specific amino acids in the C- and N-terminal domains of RCHY1 have been identified that interact with one of its ubiquitination targets (Sheng et al., 2008), but these are not conserved in plants. Plants have an additional 3–4 homologues of RCHY1, for example MYB30-INTERACTING E3 LIGASE 1 (MIEL1) in Arabidopsis was shown to ubiquitinate the transcription factors MYELOBLASTOSIS (MYB) 30 and MYB96 (Marino et al., 2013; Lee and Seo, 2016).

In summary, bioinformatics analyses show that the domain structure of HRZ/BTS/BTSL is conserved in the green lineage. In dicotyledons, the BTS and BTSL proteins belong to separate phylogenetic clades with differences in the second Hr motif and the Zn-finger domain.

## Expression Patterns and Mutant Studies

Expression behaviour and mutant phenotypes provide evidence that *HRZ1* and *HRZ2* in rice, *BTS* in several plant species and the two redundant *BTSL* genes in Arabidopsis play comparable roles in Fe homeostasis, acting as negative regulators.

Microarray analysis of root tissues showed that *BTS* is up-regulated as early as 12–24 h after transferring seedlings to medium lacking Fe, especially in the root stele above the differentiation zone (Dinneny et al., 2008; Long et al., 2010). Further expression analysis of shoots and roots showed that *BTS* transcripts are much more abundant in shoots than in roots (Rodríguez-Celma et al., 2013; Hindt et al., 2017). Initial promoter-GUS studies in young seedlings showed no Fe-dependent regulation of *BTS* in the leaves (Selote et al., 2015), contrasting with RNAseq data of >10-fold induction under Fe deficiency (Rodríguez-Celma et al., 2013; Hindt et al., 2017). Possibly, sequences outside the cloned promoter region may contribute to the transcriptional regulation of *BTS*, but further investigation is needed.

Rice *HRZ1* and *HRZ2* are also induced within 1 day after transfer to Fe-deficient medium, and transcript levels increase further over the next 6 days without Fe (Kobayashi et al., 2013). Both *HRZ* paralogs are expressed in roots and shoots, but about 10 times higher in shoots (Kobayashi et al., 2013).

The expression of *BTSL1* and *BTSL2* in Arabidopsis is restricted to the roots under Fe deficiency (Hindt et al., 2017; Rodríguez-Celma et al., 2017). Promoter-GUS studies revealed expression in the root hairs, epidermis, cortex and endodermis (Rodríguez-Celma et al., 2017). The *BTSL* genes are coregulated with the root ferrome (Schmidt and Buckhout, 2011), suggesting common upstream transcription factors.

*BTSL2* was de-regulated in the *fit-1* and *fit-3* mutants lines, which are defective in FER-LIKE IRON DEFICIENCY INDUCED TRANSCRIPTION FACTOR (FIT), the main regulator of Fe uptake in dicotyledonous plants (Colangelo and Guerinot, 2004; Jakoby et al., 2004). *HRZ1* and *HRZ2* expression was transiently enhanced in lines overexpressing the *IDEF1*, the IRON-DEFICIENCY-RESPONSIVE ELEMENT (IDE) BINDING FACTOR 1, but not *IDEF2* (Kobayashi et al., 2013). Overall, Fe deficiency affects the expression levels of Arabidopsis *BTS* and *BTSL* genes more than any other stress condition (Supplementary Table 1^1^).

Knockout alleles of *BTS* are embryo lethal (McElver et al., 2001; Selote et al., 2015), but lines with a T-DNA insertion in the promoter are viable despite lower seed production (Long et al., 2010; Selote et al., 2015). The mutants accumulate Fe in the whole plant but especially in the seeds which is the likely cause of embryo lethality. Viable *bts* mutants also showed increased acidification of the rhizosphere and ferric-chelate reductase activity under Fe deficiency (Selote et al., 2015). Recently, a new allele of *BTS* was found in a mutant screen for altered element profiles (Hindt et al., 2017). The *bts-3* allele carries a missense mutation affecting the second Zn-finger of the RING motif (Pro1174Leu). *bts-3* plants accumulated more Fe than the promoter mutant *bts-1* (244 and 32% over wild-type, respectively) and growth of *bts-3* was severely compromised in the presence of Fe.

In contrast to *BTS*, knockout mutants of Arabidopsis *BTSL* are viable (Hindt et al., 2017; Rodríguez-Celma et al., 2017). Phenotypes are only observed in the *btsl* double mutant, which retained more chlorophyll than wild type under Fe deficiency and has increased Fe levels in both roots and shoots. A triple mutant with *bts-1* displayed an enhanced phenotype (Hindt et al., 2017).

To study the function of the rice *HRZ* genes, RNAi lines and insertion mutants have been generated. Two different *hrz1* alleles, *hrz1-1* with decreased transcript levels and *hrz1-2* with a frameshift predicted to remove the E3 ligase domain, were more tolerant to Fe deficiency than wild type, accumulated Fe in the seeds and had lower grain yield (Kobayashi et al., 2013; Zhang et al., 2017). For *HRZ2*, RNAi lines and the *hrz2-1* mutant also showed tolerance to Fe deficiency and accumulated Fe in leaves and seeds (Kobayashi et al., 2013). Interestingly, detailed phenotypic and gene expression studies of the mutants showed that the *HRZ* genes also have a function in Fe sufficiency and excess (Zhang et al., 2017; Aung et al., 2018).

The *BTS* homologue in the legume *Lotus japonicus* is strongly upregulated in developing root nodules (Shimomura et al., 2006), perhaps correlated with the high demand for Fe in the plant-bacterial symbiosis (Brear et al., 2013). RNAi silencing of the gene resulted in chlorosis, retarded growth and roots which failed to nodulate. The growth phenotypes could not be rescued by exogenous nitrogen supply, which usually overcomes a lack of symbiotic nitrogen fertilisation. However, Fe-dependent regulation and phenotypes have not been investigated to date. Another report identified the BTS homologue in tobacco, *Nicotiana tabacum*, in the context of tobacco mosaic virus infection (Yamaji et al., 2010).

To sum up, *HRZ*/*BTS*(*L*) transcript levels are strongly induced under Fe deficiency, and mutant lines have an enhanced or constitutive response to Fe deficiency, resulting in Fe accumulation in all tissues.

## Metal Binding, Protein Stability and Localisation

The HRZ/BTS/BTSL proteins are predicted to bind metals both in the Hr motifs and the Zn-fingers. Hemerythrins bind 2 Fe ions, thus HRZ and BTS are predicted to bind a total of 6 Fe in their 3 Hr motifs, and BTSLs are likely to bind 4 Fe. Conservation of the Zn-binding sites in the C-terminal domain of HRZ/BTS proteins compared to RCHY1/Pirh2 suggests 9 Zn ions are required for structural integrity (Sheng et al., 2008). Experimental data for HRZ and BTS to date falls well short of these numbers. Full-length recombinant BTS purified from *Escherichia coli* was found to bind 2 Fe and 5 Zn (Selote et al., 2015). Full length HRZ1 co-purified with 2 Fe and 1 Zn, and the truncated version of the HRZ2 protein bound 2 Fe and 2 Zn ions (Kobayashi et al., 2013). Despite the lower than expected number of Zn ions binding to the E3 ligase domain, the recombinant HRZ and BTS proteins had ubiquitin transferase activity, evident from self-ubiquitination (Kobayashi et al., 2013; Selote et al., 2015; Zhang et al., 2017). Most likely, a small proportion of HRZ/BTS protein had a full complement of Zn ions among mostly apo-protein.

The HRZ/BTS/BTSL proteins appear highly unstable. Attempts to detect the endogenous protein *in vivo* have been unsuccessful to date, and even full-length GFP fusions of HRZ or BTS are difficult to detect either by western blot analysis or fluorescence microscopy (Kobayashi et al., 2013; Selote et al., 2015). Interestingly, a truncated version of BTS lacking the Hr domain is easily detectable in roots and complements *bts-1* phenotypes (Selote et al., 2015). This suggests that the Hr domain may contribute to the instability of HRZ/BTS proteins. Furthermore, root extracts from rice (but not shoot extracts) were able to degrade recombinant HRZ1 and HRZ2 in a MG132-dependent manner, pointing at proteasomal degradation following self-ubiquitination or ubiquitination by other E3 ligases (Kobayashi et al., 2013; Zhang et al., 2017).

The HRZ/BTS proteins localised to the nucleus when full-length GFP fusions were transiently expressed in onion cells and *Nicotiana benthamiana*, respectively (Kobayashi et al., 2013; Selote et al., 2015). No data is yet available about the localisation of BTSL proteins, but they are presumably also nuclear. Interestingly, a truncated version of BTS lacking the Hr domain but preserving the first N-terminal amino acids was localised to the cytosol (Selote et al., 2015). Truncated versions of BTS containing only the Hr motifs or lacking the RING motif were localised to both the cytosol and the nucleus (Selote et al., 2015). A similar observation was made for HRZ2 in Kobayashi et al. (2013), using a truncated version of HRZ2 containing only the third hemerythrin motif and the C-terminal E3 ligase domain.

How does Fe binding to the Hr motifs relate to protein stability? This key question has been addressed using transient expression of BTS-GFP in *N. benthamiana* leaves and by using a wheat germ *in vitro* translation system (Selote et al., 2015). BTS-GFP fluorescence was only observed when deferoxamine, an Fe chelator, was co-infiltrated. Addition of micromolar amounts of Fe to the wheat germ extract prevented accumulation of BTS protein, but it was produced upon deletion of the entire Hr domain. Selective mutagenesis of putative Fe-binding ligands in the first or second Hr motif, but not the third, also stabilised the BTS protein (Selote et al., 2015). Thus, these data suggest that BTS is destabilised by Fe binding to the Hr motifs, in contrast to the mammalian FBXL5 protein, which is stabilised by Fe binding. Given there are 3 Hr domains in the plant proteins, Fe binding properties are likely to be more complex than a simple de/stabilisation effect, as discussed below.

Thus, the data obtained by different experimental approaches suggest that the abundance and subcellular localisation of HRZ/BTS/BTSL proteins are tightly controlled, regulated by Fe binding to the N-terminal Hr domain and self-ubiquitination.

## Interaction Partners

A yeast-2-hybrid screen with BTS as bait against a root-specific cDNA library identified several potential interaction partners, including the basic Helix-Loop-Helix (bHLH) transcription factors bHLH104 and bHLH115 which are involved in Fe homeostasis (Long et al., 2010). *In planta* bimolecular fluorescence complementation (BiFC) and co-immunoprecipitation confirmed that BTS interacts with bHLH104, bHLH115, and also with bHLH105 (ILR3), but not with the bHLH protein POPEYE (PYE) (Selote et al., 2015). Further investigation using an *in vitro* cell-free degradation assay with or without the inhibitor MG132 indicated that BTS mediates the degradation of ILR3 and bHLH115 (Selote et al., 2015).

A similar yeast-2-hybrid screen using rice HRZ1 identified the bHLH transcription factor POSITIVE REGULATOR OF IRON HOMEOSTASIS 1 (PRI1) as interaction partner, which is the homologue of Arabidopsis ILR3 (Zhang et al., 2017). Co-localisation of transiently produced HRZ1 and PRI1 in the nucleus and co-immunoprecipitation further supported the interaction (Zhang et al., 2017). PRI1 promotes the expression of *IRO2* and *IRO3*, which are orthologs of *bHLH38/39* and *PYE*, respectively (Kobayashi and Nishizawa, 2012). Interestingly, the homology of the interaction partners in Arabidopsis and rice suggest that this part of the Fe-regulatory circuit is conserved in dicot and monocot species.

Protein interaction screens have so far not been reported for the BTSL proteins but phenotypic studies have provided some clues. When the *btsl* double mutant was exposed to Fe-deficiency and resupply treatments, a specific pattern of mis-regulated expression of the Fe uptake genes *FERRIC REDUCTASE OXIDASE 2* (*FRO2)* and *IRON-REGULATED TRANSPORTER 1* (*IRT1)* suggested that FIT, or a transcription factor further upstream, is a target for degradation by BTSLs (Rodríguez-Celma et al., 2017). Previous studies by Sivitz et al. (2011) found that FIT protein levels are controlled by proteasomal degradation, but the E3 ligase had not been identified. Physical interaction between BTSL1/2 and FIT has been shown *in vitro* using far-Western blot analysis (Rodríguez-Celma et al., 2017). FIT functions as a heterodimer with bHLH38/39, the expression of which is controlled by ILR3 and bHLH104 (Zhang et al., 2015; Li et al., 2016). Interestingly, mis-regulation of *bHLH38/39* expression is also found in the *btsl* double mutant (Rodríguez-Celma et al., 2017). This suggests that BTSLs may have additional targets such as ILR3 and/or bHLH104.

Very recently, it was shown that BTS interacts with another set of transcription factors, VASCULAR PLANT ONE-ZINC FINGER (VOZ) 1 and VOZ2 (Selote et al., 2018). VOZ1 and VOZ2 negatively regulate drought and cold responses, and VOZ2 has previously been shown to be degraded by the 26 proteasome. VOZ1/2 are primarily present in the cytosol, but likely translocate to the nucleus through phosphorylation to interact with BTS (Yasui et al., 2012; Koguchi et al., 2017; Selote et al., 2018). Importin (IMP) α-4. Localised in the nucleus, interacts with BTS but is not a target for degradation (Selote et al., 2018). This suggests that IMPα-4 may play a role in nuclear localisation of BTS and BTS-interacting proteins.

These findings suggest HRZ/BTS/BTSL might be involved in other stress conditions that are sensitive to Fe levels, possibly due to the role of Fe in the reactive oxygen species (ROS) response (Tripathi et al., 2018). Future studies to characterise HRZ/BTS/BTSL interacting proteins will help to shed light on how these Fe sensors may integrate multiple stress responses.

## Working Model

**FIGURE 2:**
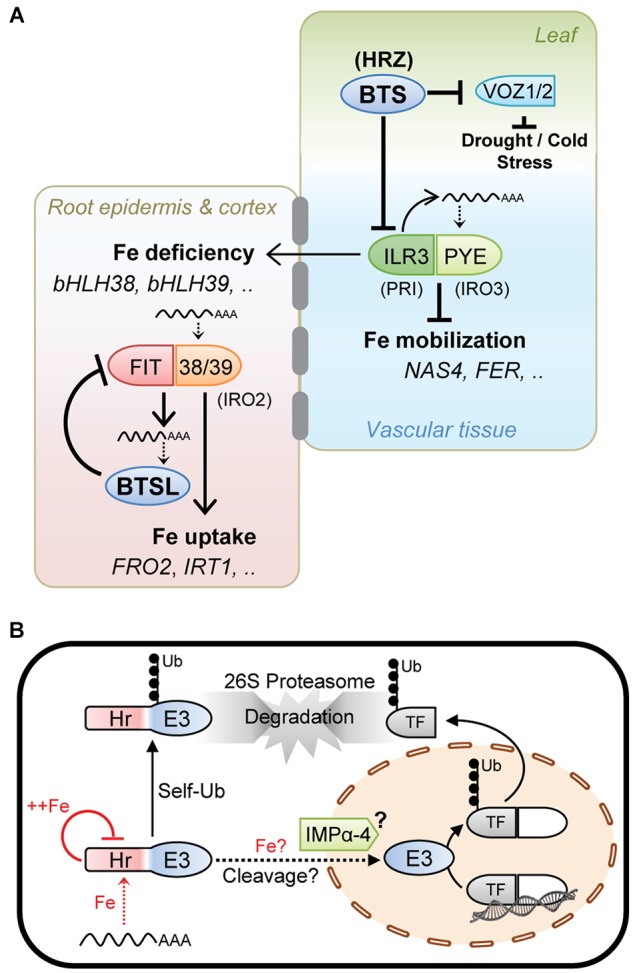
Working model of the hemerythrin E3 ligases as negative regulators of the Fe deficiency response. **(A)** HRZ/BTS/BTSL protein function integrated with the iron homeostasis regulatory cascade. The Arabidopsis gene acronyms are used, with rice orthologs in brackets. BTSL represents two mostly redundant proteins in Arabidopsis, and HRZ represents the HRZ1 and HRZ2 proteins. **(B)** Cellular mode of action of HRZ/BTS/BTSL proteins. Fe levels are thought to affect protein translation and stability. Protein stability is further controlled via self-ubiquitination activity and proteasomal degradation. HRZ/BTS have been shown to localise in the nucleus. Protein interaction data indicate that importin (IMP) α-4 may facilitate nuclear import of BTS and that the hemerythrin (Hr) domain may be cleaved off. Once in the nucleus, HRZ/BTS/BTSL catalyse the ubiquitination of specific transcription factors followed by proteasomal degradation.

Taken together, the experimental data on HRZ, BTS, and BTSL, although patchy in places, suggests a similar mode of action of these proteins, namely they provide a negative feedback loop to moderate the activity of specific transcription factors and hence limit Fe accumulation (Figure 2A).

Present in all plant species, HRZ/BTS proteins act in shoots and root vascular tissue to moderate the Fe deficiency response and Fe mobilisation (Figure 2A). The BTSL proteins are found in dicotyledonous plants only and inhibit Fe uptake. These two regulatory loops are connected through the expression of *bHLH38* and *bHLH39*, two transcription factors needed for Fe uptake whose expression is regulated by ILR3, a target of BTS (Figure 2A).

The transcript levels of *HRZ*/*BTS*/*BTSL* are strongly induced by Fe deficiency. Protein translation may depend on low amounts of Fe to allow folding of the N-terminal Hr domain (Figure 2B), as shown for FBXL5 in mammals, and to proceed with translation of the C-terminal E3 ligase domain. Because FIT is required for *BTSL* transcription and also for replenishing Fe in the cytosol, this creates a time delay in the feedback loop. Interestingly, Sivitz et al. (2011) observed that FIT protein starts being turned over ∼48 h after its upregulation in response to Fe deficiency.

On the other hand, Fe binding to the Hr domain was shown to negatively control the levels of BTS protein (Figure 2B). Possibly, once sufficient Fe is available, most of the BTS is degraded, leaving only a residual amount functioning under Fe sufficiency and excess. HRZ/BTS/BTSL protein levels would also be tightly controlled by self-ubiquitination leading to proteasomal degradation (Figure 2B).

Since truncated BTS-GFP lacking the Hr domains is stable and functionally complemented the *bts-1* mutant, it is tempting to speculate that the Hr domain is cleaved off, resulting in a ∼40 kDa protein which is small enough for nuclear import facilitated by the interaction with the importin IMPα-4 (Figure 2B). A precedent for such a relocation mechanism is provided by the Fe-regulated transcription factor Aft1 in yeast, which shuttles between the cytosol and nucleus depending on the Fe status of the cell (Outten, 2017). A possible role of Fe binding to the hemerythrin motifs in the cleavage or relocation of the protein cannot be ruled out (Figure 2B). Once in the nucleus, the E3 ligase domain would be able to interact with and ubiquitinate its target transcription factors for degradation.

From expression networks and promoter-GUS studies, it has become clear that BTS and BTSLs have diverged functions in dicotyledonous plants. *BTSLs* are expressed in root tissues that take up Fe from the soil, whereas *BTS* acts in the stele and shoots. The presence of 2 or 3 Hr motifs, respectively, suggests that they respond to different Fe setpoints on each side of the endodermis, a cell layer that performs a critical function in nutrient uptake (Barberon et al., 2016).

To further test the working model, the following research questions will need to be addressed:

• Determine how Fe binding to the Hr domain alters the stability and localisation of the HRZ/BTS/BTSL proteins, and whether this domain is removed for nuclear import. For this, detection of the endogenous proteins will be key.
• Identify possible phosphorylation sites that could affect nuclear import.
• Investigate if the di-iron cofactors are redox active, and whether the HRZ/BTS proteins are modulated by oxygen levels, as is the case for FBXL5.
• Test the “different Fe setpoint hypothesis” for roots and shoots by swapping the Hr domains of BTS and BTSLs and expressing them in the respective mutants.
• In dicots, investigate possible connexions between the BTS and BTSL feedback loops, for example through the expression of bHLH38/39.
• Identify the transcription factors that regulate *BTS* expression, and confirm those that are likely to regulate the *HRZ* and *BTSL* genes.

## Author Contributions

All authors contributed to the writing of the manuscript, including discussion, and proofreading.

## Conflict of Interest Statement

The authors declare that the research was conducted in the absence of any commercial or financial relationships that could be construed as a potential conflict of interest.

## Footnotes

1 www.araport.org
